# Biomaterial-based Drug delivery strategies for head and neck cancer: Local delivery, combination therapy, and translational challenges

**DOI:** 10.1016/j.ijpx.2026.100651

**Published:** 2026-08-26

**Authors:** Chang Li, Yuan Liu, Zhifeng Wen, Junxiong Wen, Xiao Cui, Xue Zhang

**Affiliations:** a Department of Stomatology, The Fourth Affiliated Hospital of China Medical University, Shenyang, China; b Department of Neurosurgery, The First Hospital of China Medical University, Shenyang, China; c Department of Otolaryngology Head and Neck Surgery, Shengjing Hospital of China Medical University, Shenyang, China; d Department of Otorhinolaryngology Head and Neck Surgery, The First Hospital of China Medical University, Shenyang, China; e The VIP Department, School and Hospital of Stomatology, China Medical University, Shenyang, China

**Keywords:** Head and neck cancer, Head and neck squamous cell carcinoma, Biomaterials, Drug delivery, Combination therapy

## Abstract

Head and neck cancer, particularly head and neck squamous cell carcinoma, remains difficult to treat because of local recurrence, treatment-related morbidity, and the limited efficacy of systemic drug delivery. Biomaterial-based delivery systems have emerged as useful tools to improve local drug retention, control release kinetics, and support combination treatment. Hydrogels, nanoparticles, liposomes, microneedles, and implantable systems have been explored for the delivery of chemotherapeutic agents, radiosensitizers, immunotherapeutics, and nucleic acids in head and neck cancer. This review summarizes recent progress in biomaterial-based drug delivery strategies, with a focus on local delivery and combination therapy. We discuss the major biomaterial platforms, their therapeutic applications, and the practical challenges that affect clinical translation, including tumor heterogeneity, dosing reproducibility, biocompatibility, and manufacturability. Overall, biomaterials represent a promising but still developing approach for improving treatment delivery and multimodal therapy in head and neck cancer.

## Graphical Abstract

**Figure:**
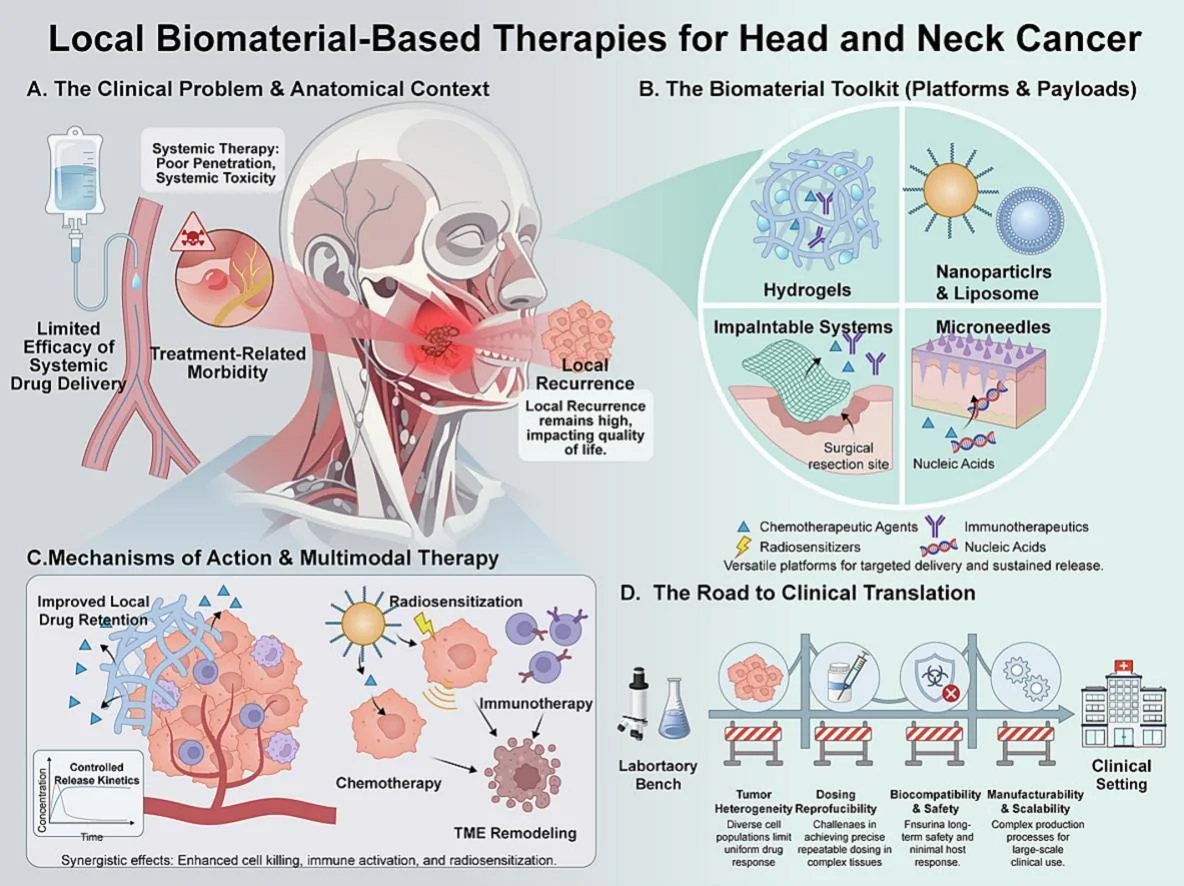

## 1 Introduction

Cancer is a complex group of diseases responsible for the deaths of millions of people annually (Gilbertson, 2011). The metastatic behavior of tumor cells varies depending on the origin of the cancer and the organ they affect. Head and neck cancer (HNC) encompasses a diverse group of malignancies originating from the oral cavity, pharynx, larynx, nasal cavity, and salivary glands. Among these, head and neck squamous cell carcinoma (HNSCC) and oral squamous cell carcinoma (OSCC) represent the vast majority, accounting for approximately 90% of all cases (Johnson et al., 2020; Liao et al., 2026). Despite significant advancements in diagnostic techniques and therapeutic modalities, including surgery, radiotherapy, chemotherapy, and targeted therapies, the prognosis for advanced-stage HNC remains poor, with a 5-year survival rate hovering around 50–60% (Johnson et al., 2020; Tang et al., 2024). The persistent challenges in HNC treatment stem from several inherent complexities associated with the disease and its anatomical location, which critically underscore the urgent need for innovative drug delivery strategies, particularly those leveraging biomaterials.

The complex local anatomy of the head and neck region presents formidable obstacles to effective drug delivery (Feng et al., 2026, Li et al., 2026, Liao et al., 2024, Liao et al., 2025). This area is characterized by a dense network of vital structures, including major blood vessels, nerves, and airways, all in close proximity to the tumor. Systemic administration of therapeutic agents often leads to insufficient drug accumulation at the tumor site due to rapid clearance, poor vascularization within the tumor, and the presence of various biological barriers (Liao et al., 2026, Lin et al., 2025, Pan et al., 2025, Shi et al., 2025). Moreover, achieving precise local delivery without damaging adjacent healthy tissues is exceptionally challenging, making conventional approaches less effective and more prone to off-target effects (Wu and Zhou, 2015; Del Campo-Balguerías et al., 2025). Biomaterials offer a unique opportunity to overcome these anatomical constraints by enabling highly localized and sustained drug release directly at the tumor site, thereby maximizing therapeutic concentration while minimizing systemic exposure.

The mucosal barriers and the salivary environment significantly impact drug delivery, particularly in oral and oropharyngeal cancers. The oral mucosa, while permeable to some extent, acts as a protective barrier, limiting the penetration of topically applied drugs. Furthermore, the continuous flow and enzymatic activity of saliva can rapidly dilute, degrade, and clear therapeutic agents, reducing their residence time and bioavailability at the target site (Ketabat et al., 2019). This dynamic environment necessitates drug delivery systems that can adhere to mucosal surfaces, resist salivary washout, and facilitate controlled drug release over an extended period. Biomaterial-based platforms, such as mucoadhesive hydrogels or patches, are specifically designed to address these challenges, offering enhanced drug retention and improved therapeutic efficacy in the oral cavity.

The significant toxicity associated with conventional systemic therapies remains a major concern in HNC management (Liao et al., 2024, Liao et al., 2026, Liu et al., 2025). High doses of chemotherapeutic agents like cisplatin and 5-fluorouracil, while effective, often lead to severe systemic side effects, including nephrotoxicity, ototoxicity, myelosuppression, and mucositis, which can significantly impair patients' quality of life and necessitate dose reductions or treatment interruptions (Johnson et al., 2020; Mohit et al., 2026). Similarly, radiation therapy (RT), a cornerstone of HNC treatment, can cause debilitating side effects such as xerostomia, dysphagia, and osteoradionecrosis. The ability of biomaterials to localize drug delivery to the tumor, thereby reducing systemic drug concentrations, holds immense promise for mitigating these adverse effects and improving the therapeutic index of existing treatments (Del Campo-Balguerías et al., 2025; Srinivasan and Prasad, 2025).

The growing demand for combination therapies in HNC reflects the complex and heterogeneous nature of the disease. HNC often involves multiple oncogenic pathways, and single-agent therapies frequently encounter drug resistance or insufficient efficacy (Liao et al., 2026, Wang et al., 2025, Wu et al., 2026, Xiao et al., 2024, Xie et al., 2025, Yang et al., 2023). Multimodal approaches, combining chemotherapy, radiotherapy, targeted therapy, and immunotherapy, are becoming standard practice to achieve synergistic effects and overcome resistance mechanisms (Johnson et al., 2020; Liang et al., 2021). Biomaterials are uniquely positioned to facilitate these complex combination strategies by enabling the co-delivery of multiple therapeutic agents with distinct release profiles, sensitizing tumors to other treatments, or modulating the tumor microenvironment (TME) to enhance immune responses. The typical biomaterials in use are composed of proteins, polysaccharides, lipids, and other degradable materials (Liang et al., 2021). For instance, nanoparticles can encapsulate various drugs, while hydrogels can serve as scaffolds for sustained co-delivery, opening new avenues for highly effective and personalized combination regimens (Tan et al., 2020; Zou et al., 2025; Yan et al., 2020). Biomaterial-based implants or injectable systems that can deliver anticancer agents directly into the surgical bed or surrounding tissues offer a compelling solution for localized, controlled release over weeks or months, potentially improving local control and patient outcomes (Tan et al., 2020; Li et al., 2019a).

In light of these multifaceted challenges, biomaterial-based drug delivery strategies have emerged as a transformative approach in HNC research. This review aims to provide a comprehensive overview of recent advancements in this field, organizing the discussion around clinical application scenarios rather than simply by material type. We will delve into how specific biomaterial platforms enhance drug retention, controlled release, tissue penetration, and compatibility with various combination therapies. Furthermore, a dedicated section will address the critical translational challenges, rigorously distinguishing between preclinical findings and early clinical explorations, and highlighting the factors that currently constrain the clinical applicability of these innovative strategies. The primary focus will be on HNSCC and OSCC, reflecting the predominant evidence base in the literature.

## 2 Localized/Intratumoral delivery

Localized or intratumoral drug delivery represents a highly attractive strategy for HNC due to the direct accessibility of many tumor sites and the potential to achieve high drug concentrations within the tumor while minimizing systemic exposure and associated toxicities (Huang et al., 2020; Harrington, 2025). Biomaterial-based systems are particularly well-suited for this approach, as they can be engineered to enhance drug retention, control release kinetics, and improve tissue penetration within the complex TME. The TME, characterized by abnormal vasculature, dense extracellular matrix (ECM), hypoxia, and altered pH, often impedes the effective distribution of systemically administered drugs (Feng et al., 2024; Sleeboom et al., 2024; Prakash and Shaked, 2024). Biomaterials can be designed to overcome these barriers, offering a more precise and potent therapeutic intervention.

### 2.1 Nanoparticle-based Intratumoral delivery

**Fig. 1:**
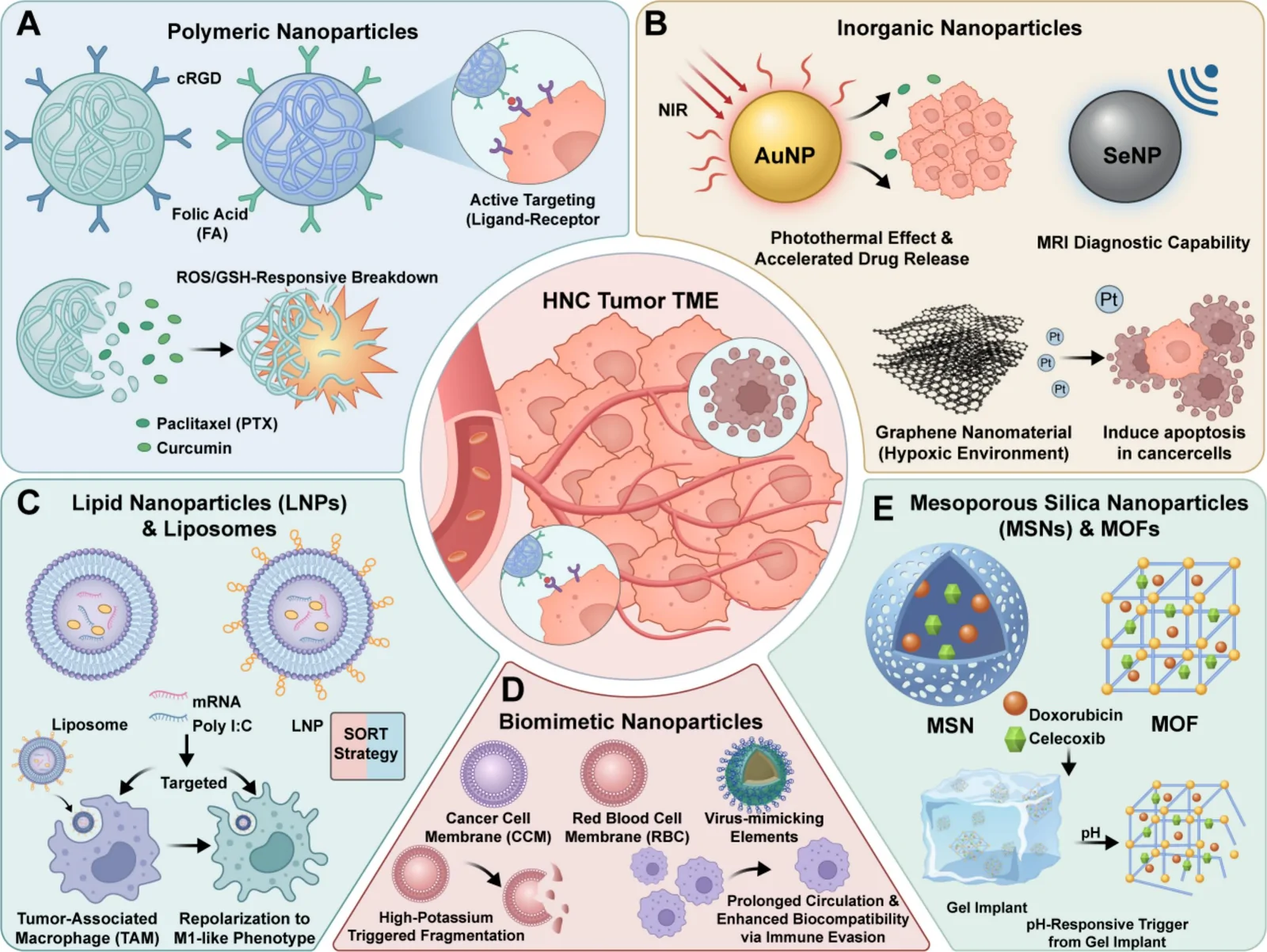
Diverse Nanoparticle Platforms for Intratumoral Delivery and Multimodal Therapy in Head and Neck Cancer. Diverse nanoparticle platforms for intratumoral delivery and multimodal therapy in head and neck cancer (HNC). The central panel illustrates the complex HNC tumor microenvironment (TME), characterized by abnormal vasculature and dense extracellular matrix, where nanoparticles exhibit enhanced penetration and targeted accumulation compared to conventional formulations. (A) Polymeric nanoparticles can be engineered with targeting ligands (e.g., cRGD, folic acid) and stimuli-responsive breakdown mechanisms (e.g., ROS or GSH) for the precise release of chemotherapeutics such as paclitaxel or curcumin. (B) Lipid nanoparticles (LNPs) and liposomes facilitate the delivery of nucleic acids (e.g., mRNA, Poly I:C) and can repolarize tumor-associated macrophages (TAMs) to an anti-tumor M1-like phenotype, guided by Selective Organ Targeting (SORT) strategies. (C) Inorganic nanoparticles, including gold (AuNPs), selenium (SeNPs), and graphene-based nanomaterials, enable synergistic modalities such as photothermal therapy (PTT), MRI diagnostics, and hypoxia-targeted apoptosis. (D) Mesoporous silica nanoparticles (MSNs) and metal-organic frameworks (MOFs) offer high surface areas and tunable pores for high drug loading (e.g., Doxorubicin and Celecoxib) and pH-responsive release within gel implants. (E) Biomimetic nanoparticles coated with cancer cell, red blood cell, or virus-mimicking membranes ensure prolonged circulation, immune evasion, and triggered fragmentation in specific physiological states. (For interpretation of the references to colour in this figure legend, the reader is referred to the web version of this article.)

Nanoparticles (NPs) are a versatile class of biomaterials extensively explored for intratumoral delivery due to their tunable size, surface properties, and ability to encapsulate diverse therapeutic payloads (Del Campo-Balguerías et al., 2025; Srinivasan and Prasad, 2025; Li et al., 2024a; Gül et al., 2024). Their small size allows for better penetration into tumor tissues compared to larger conventional drug formulations, and their modifiable surfaces enable active targeting and stimuli-responsive release (Fig. 1).

#### 2.1.1 Polymeric nanoparticles

Polymeric nanoparticles, composed of natural or synthetic polymers, are widely investigated for their biocompatibility, biodegradability, and capacity for controlled drug release (Srinivasan and Prasad, 2025). For instance, paclitaxel (PTX), a potent chemotherapeutic agent, faces challenges due to its poor solubility and systemic toxicity (Muley et al., 2025). To address this, paclitaxel-loaded ROS-responsive nanoparticles (PTX@TPGS-PBTE NPs) were developed, demonstrating enhanced cytotoxicity and apoptosis in SCC-7 cells in vitro (Tu et al., 2023). The incorporation of cRGD modification on these nanoparticles further improved tumor-targeted delivery and significantly inhibited tumor growth in SCC-7 murine models, highlighting the advantage of active targeting in enhancing therapeutic effects against HNC (Tu et al., 2023). Similarly, the co-delivery of paclitaxel and curcumin (Cm) in PLGA nanoparticles, optimized using a Box–Behnken design, resulted in nanoparticles with an average size of approximately 100 nm and high encapsulation efficiencies for both drugs (Hu et al., 2023). These nanoparticles exhibited enhanced cytotoxicity with lower IC50 values against breast cancer cell lines compared to free drugs, suggesting their potential for HNC, given the common challenges of drug resistance and toxicity.

Another innovative approach involves glutathione (GSH)-sensitive and folic acid (FA)-targeted nanoparticles loaded with paclitaxel (FA-PEG-S-S-PCL@PTX, FA-NPs) for OSCC therapy (Fan et al., 2020a). Given that FA receptors are expressed in a high percentage (95%) of OSCC specimens, these FA-NPs enhanced FA-mediated endocytosis in HSC3 cells, leading to increased accumulation at tumor sites and superior antitumor effects compared to free PTX and non-targeted nanoparticles in preclinical models^24^. This demonstrates the power of combining stimuli-responsiveness with active targeting to improve drug delivery specificity and efficacy.

#### 2.1.2 Lipid nanoparticles and liposomes

Lipid nanoparticles (LNPs) and liposomes are well-established platforms for drug delivery, known for their biocompatibility and ability to encapsulate both hydrophilic and hydrophobic drugs, as well as nucleic acids (Liao et al., 2026; Dhiman and Sharma, 2024). In the context of HNC, LNPs have shown significant promise, particularly for gene therapy and immunotherapy. For example, a novel LNP (LNPHNSCC) was identified through high-throughput screens for its ability to preferentially deliver mRNA to HNSCC solid tumors in vivo, while minimizing off-target delivery to the liver (Huayamares et al., 2023). This selective organ targeting (SORT) strategy is crucial for enhancing the safety and efficacy of mRNA-based therapies, including CRISPR-Cas gene editing, by ensuring tissue-specific delivery (Cheng et al., 2020). Further studies have demonstrated that LNPs delivering mRNA encoding a prodrug-activating bacterial enzyme (PNP) can lead to anti-tumor responses when combined with fludarabine phosphate, suggesting a promising strategy for intratumoral cancer treatment (Huayamares et al., 2025). The ability of LNPs to deliver mRNA-encoded activators for robust and durable gene activation in vivo further expands their potential for HNC therapy (Beyersdorf et al., 2022).

For immunotherapy, a lipid nanoparticle (LNP) co-delivering p53 mRNA and ciclopirox (CPX) has been developed for oral cancer chemoimmunotherapy. This system not only exhibits chemotherapeutic properties by activating caspase but also effectively repolarizes tumor-associated macrophages (TAMs) to M1-like phenotypes, thereby reducing immunosuppression within the TME. This comprehensive approach addresses multiple mechanisms of OSCC progression and demonstrates efficacy in both p53-therapy-sensitive and -resistant models, highlighting the versatility of LNPs in multimodal therapy.

Liposomes encapsulating double-stranded nucleic acid (Poly I:C) have also been explored for HNC treatment (Yang et al., 2024, Zang et al., 2025, Zhang et al., 2025, Zhu et al., 2026, Liu et al., 2026). These liposomes effectively deliver Poly I:C to HN12 cells, activating MDA5, RIG-I, and TLR3 pathways, which leads to upregulation of caspase-3, caspase-8, and IRF3, and significant tumor growth inhibition in J:NU mouse models (Singh et al., 2024). This approach leverages the immune-stimulatory properties of Poly I:C, delivered precisely to the tumor site. Furthermore, aptamer-functionalized liposomes have been developed for enhanced targeted delivery of imiquimod in HNC, particularly for HPV-related tongue cancer (Lopes-Nunes et al., 2024). These AT11 IQ liposomes selectively targeted tongue cancer cells, reducing cell viability, proliferation, and increasing cell death, while also decreasing migration and invasion, showcasing the benefits of active targeting for improved anticancer potential (Lopes-Nunes et al., 2024).

#### 2.1.3 Inorganic nanoparticles (Gold, Selenium, Graphene, MXene, Manganese)

Inorganic nanoparticles offer unique physical and chemical properties that can be exploited for HNC therapy, including photothermal effects, radiosensitization, and imaging capabilities (Wang et al., 2024; Hang et al., 2024). Gold nanoparticles (AuNPs) are particularly notable for their plasmonic properties, enabling photothermal therapy (PTT) and enhancing radiation effects (Hang et al., 2024). A hyaluronic acid (HA)-altered gold complex delivery system (HA/Au-PDA@CZT) has been fabricated for HNSCC therapy, demonstrating high antitumor efficacy and low in vivo toxicity (He et al., 2024a). This nanodrug delivery system targets tumors via pH and near-infrared (NIR) dual stimuli. The HA coating enhances tumor-targeting through recognition and internalization mediated by CD44 receptors, while NIR radiation accelerates drug release and induces photothermal effects, leading to significant tumor volume reduction in vivo when combined with chemotherapy (He et al., 2024a). After HA-CD44 binds, it triggers grid protein-dependent or non-dependent endocytosis pathways. CD44 receptors aggregate and migrate to regions with Clathrin-coated pits or Caveolae, encapsulating nanoparticles to form endocytic vesicles and transporting them into the interior of the cell (Wang and Bourguignon, 2011). This mechanism, together with the photothermal/photodynamic performance of Au-PDA and the chemotherapy effect of CZT, forms a closed loop for the system to achieve efficient anti-tumor efficacy. Similarly, a multifunctional gold nanoplatform (PDPN Ab- and DOX-conjugated AuNPs) was developed for combined chemo- PTT against oral cancer. This system showed low toxicity, high drug loading, and enhanced antitumor efficacy both in vitro and in vivo, leveraging active targeting via PDPN antibodies (Liu et al., 2020). Core-satellite mesoporous silica‑gold nanocomposites (MSN–Fe–AuNPs) have also been designed for biological stimuli-triggered multimodal cancer therapy, exhibiting pH-dependent photothermal properties, pH-triggered drug release, and H_2_O_2_-induced radical generation, leading to complete tumor inhibition in mouse models (Jin et al., 2018).

Selenium nanoparticles (SeNPs) have also been engineered for HNC. Highly uniform and stable Se-5Fu-Gd-P(Cet/YI-12) nanoparticles were designed for nasopharyngeal carcinoma (NPC) diagnosis and therapy, combining EGFR targeting and TME-responsive abilities (Huang et al., 2019). These nanoparticles exhibited excellent MRI capability and increased intracellular uptake in NPC cells, enhancing penetration and inhibiting cell growth, invasion, and migration, demonstrating the potential for theranostic applications (Huang et al., 2019). MRI signals originate from the magnetic resonance of water molecules, hydrogen protons (^1^H) in tissues. In the presence of Gd^3+^, its powerful local magnetic field fluctuations, through dipole-dipole interactions, significantly accelerate the process of protons in surrounding water molecules returning from the excited state to the ground state (longitudinal relaxation), that is, shorten the T1 relaxation time. The theoretical basis of this process is described by the Solomon-Bloembergen-Morgan (SBM) theory (Wu et al., 2024a). The Se-5FU-Gd-P (Cet/YI-12) system was mainly used as a T1-weighted positive contrast agent, exhibiting strong paramagnetism due to seven unpaired electrons of Gd^3+^ (Yang et al., 2020).

Graphene-based nanomaterials (GBNs) are being explored for their high surface area, photothermal properties, and loading capacity (He et al., 2024b). A Pt-loaded nanocomposite based on graphene quantum dots (GPt) has been developed for drug delivery, sensitizing OSCC cells to treatment in both normoxic and hypoxic conditions (Wei et al., 2018). GPt enhanced Pt accumulation, leading to increased S phase cell cycle arrest and apoptosis, and exhibited strong inhibitory effects on tumor growth with less systemic toxicity in OSCC xenograft mice, effectively combating hypoxia-induced chemoresistance (Wei et al., 2018).

MXene biomaterials, with their unique intrinsic properties, are gaining attention in regenerative medicine and potentially in cancer therapy due to their conductivity and photothermal properties (Zhang et al., 2025a). While direct applications in HNC drug delivery are still emerging, their potential for enhancing cell interactions and local therapeutic effects is noteworthy. Manganese (Mn) nanomaterials are also promising for tumor diagnosis and therapy due to their low cost, non-toxicity, and versatile oxidation states (Huang et al., 2024). They are being investigated for drug delivery, TME regulation, and synergistic therapies, including immunotherapy, offering new avenues for HNC treatment (Huang et al., 2024).

#### 2.1.4 Mesoporous silica nanoparticles and metal-organic frameworks

Mesoporous silica nanoparticles (MSNs) are attractive due to their high surface area, tunable pore size, and excellent biocompatibility, allowing for high drug loading and controlled release (Yan et al., 2020; Li et al., 2021). Charge-reversal biodegradable MSNs have been developed for synergistic chemo/photothermal and visualized therapy, exhibiting pH-responsive charge reversal, photothermal, and biodegradation properties (Li et al., 2021). These MSNs demonstrated excellent tumor enrichment and penetration, enhancing therapeutic efficacy with MRI contrast for monitoring, suggesting clinical application potential (Li et al., 2021). Chitosan-capped pH-responsive hollow MSNs (HMSN-GM-CS-FA) co-deliver pheophorbide a (PA) and doxorubicin (DOX), combining photothermal, photodynamic, and chemotherapies for synergistic antitumor efficacy in a single formulation (Yan et al., 2020). Although this platform integrates the modalities of photothermal, photodynamic and chemotherapy, it should be strictly classified as the OSCC local treatment combined platform based on the route of administration. Its core value lies in taking advantage of the mucosal adhesion property of chitosan to the originally potent combination regimen to retain the multi-mechanism synergistic anti-tumor efficacy. If changed to intravenous injection, chitosan is prone to non-specifically bind to serum proteins in the blood, leading to rapid clearance, complement activation and even the risk of pulmonary embolism (Kang et al., 2025). A genetically designed biomolecular capping system for MSNs enables receptor-mediated cell uptake and controlled drug release, successfully delivering and releasing Actinomycin D to KB cells, offering a precise and modular theranostic platform (Datz et al., 2016).

Metal-organic frameworks (MOFs) are crystalline porous materials with high drug loading capacity and tunable structures. A multifunctional MOF-based nanohybrid combining Dox and celecoxib (Cel) named Dox/Cel/MOFs@Gel was developed as an injectable implant platform for synergistic oral cancer therapy (Tan et al., 2020). This nanohybrid showed high drug loading, pH-responsive release, and potent antitumor activity against KB and SCC-9 cells in vitro, and induced tumor apoptosis and regulated angiogenesis with reduced systemic toxicity in vivo, making it a promising candidate for local oral cancer treatment (Tan et al., 2020).

#### 2.1.5 Biomimetic nanoparticles

Biomimetic nanoparticles, which mimic biological entities like cells or viruses, offer enhanced biocompatibility, prolonged circulation, and improved targeting capabilities. Cancer cell membrane (CCM)-shielded biomimetic nanoparticles (PAMAM/DOX@CCM, PDC NPs) have been designed for nasopharyngeal carcinoma (NPC) active-targeting therapy (Sun et al., 2024). These PDC NPs exhibited high DOX-loading and stability, strong cellular uptake via homologous targeting, and remarkable tumor-targeting and effective tumor suppression in vivo, representing a minimalist nanoplatform for precise NPC therapy (Sun et al., 2024). Another example is CD44-targeted virus-mimicking nanomedicine (PTC209@VNP-HA), which enhances the efficacy of BMI1 inhibitor in HNSCC (Chen et al., 2025). VNP-HA improved drug delivery efficiency, leading to a 12-fold increased PTC209 bioavailability, inhibiting cancer stem cells (CSCs), proliferation, and metastasis, and overcoming cisplatin resistance in murine models (Chen et al., 2025). Red blood cell membrane vesicles (RBCVs) modified with iRGD-TRP-PK1 have also been developed as a targeted chemotherapeutic delivery system for HNC (Bai et al., 2025). These RBCVs facilitated tumor homing and fragmented within the tumor cells' high‑potassium environment, releasing the drug, which suppressed tumor growth while minimizing side effects (Bai et al., 2025).

### 2.2 Hydrogel-based Intratumoral delivery

Hydrogels, three-dimensional polymeric networks capable of absorbing large amounts of water, are excellent candidates for localized drug delivery due to their biocompatibility, injectability, and ability to provide sustained drug release (Wu et al., 2026; Dilixiati et al., 2026; Hajebi et al., 2019). They can be directly injected into or around the tumor, forming a depot that slowly releases therapeutic agents.

One notable example is nano doxorubicin-indocyanine green matrix metalloproteinase-responsive hydrogel (NDIMH) synthesized for chemophototherapy in HNSCC (Wang et al., 2019a). This hydrogel showed uniform size distribution and sustained release in the presence of MMP-2, an enzyme often overexpressed in tumors. Under NIR light, NDIMH effectively inhibited SCC-15 viability, invasion, and metastasis in vitro, and intratumoral injection with NIR illumination demonstrated favorable synergistic antitumor efficacy and biosafety in vivo, with improved nanodrug retention at the tumor site (Wang et al., 2019a). Similarly, DOX encapsulated in biodegradable micelles and loaded into an in situ HA hydrogel cross-linked by an MMP-2-responsive peptide showed sensitive MMP-2-responsive drug release and high cytotoxicity against SCC-15 cells. In vivo, these HA hydrogels degraded faster in tumors, leading to local sustained DOX release and tumor growth inhibition without organ damage, proving promising for local chemotherapy (Li et al., 2019a).

Another study incorporated Erlotinib (Er) and Cm loaded nanoparticles (Er/Cm-NP) into thermosensitive chitosan hydrogels (optEr/Cm-NP-HG) for intratumoral delivery in HNSCC (Haider et al., 2024). These NP-loaded hydrogels demonstrated sustained drug release and enhanced cytotoxicity against HNSCC cells in vitro, with improved cellular uptake confirmed by confocal microscopy. In vivo studies in HNSCC mouse models showed significant tumor growth inhibition, suggesting their potential for HNSCC therapy (Haider et al., 2024).

A self-assembling RADA16-I peptide in situ hydrogel loaded with Celastrol (RADA-CeT) has been developed to boost immunogenic cell death (ICD) in OSCC (Zhang et al., 2025b). This biocompatible drug delivery system demonstrated excellent anti-tumor efficacy by amplifying ICD, highlighting its potential for OSCC immunotherapy (Zhang et al., 2025b). Furthermore, a chitosan-ZnTAPP hydrogel loaded with cisplatin (CDDP) was developed for photodynamic therapy (PDT) to overcome cisplatin resistance in solid tumors (Zhang et al., 2025c). Under irradiation, this hydrogel significantly decreased the IC50 for CDDP-resistant lung cancer cells, demonstrating its potential as a photodynamic anticancer drug delivery system to combat resistance, which is highly relevant for HNC.

### 2.3 Other implantable systems

Beyond nanoparticles and hydrogels, other implantable systems are being investigated for localized delivery. Bioactive sutures, for instance, have been explored as a novel immunotherapy for HNC (Shibuya et al., 2004). These sutures, loaded with immunomodulatory agents like IFN-γ, IL-2, and anti-CD3/CD28, induced a sustained Th1 immune response in vitro, expanding flu-specific T cells and stimulating IL-12 secretion. This approach offers a novel in situ strategy for enhancing cancer patients' immune systems, particularly in the context of surgical resection (Shibuya et al., 2004).

Intranodal drug delivery systems (LDDS) are also being developed to address the challenge of low drug delivery rates to metastatic lymph nodes, which are common in HNC (Kodama and Sukhbaatar, 2025). By injecting anticancer drugs directly into lymph nodes, this system showed promising results in preclinical mouse models, with the first clinical study initiated in Japan in 2024, indicating its potential for treating regional metastasis (Kodama and Sukhbaatar, 2025).

### 2.4 Advantages and disadvantages of localized/Intratumoral delivery

The primary advantage of localized/intratumoral delivery is the ability to achieve significantly higher drug concentrations at the tumor site compared to systemic administration, leading to enhanced therapeutic efficacy and reduced systemic toxicity (Huang et al., 2020). Biomaterials facilitate this by providing sustained release, protecting drugs from degradation, and enabling targeted delivery. For example, the redox-responsive micelles by Sun et al. achieved 10-fold selectivity against cancer cells compared to normal cells (Sun et al., 2018), while the HA/Au-PDA@CZT nanoplatform significantly reduced tumor volume with low in vivo toxicity (He et al., 2024a). The use of stimuli-responsive systems, such as pH-responsive nanoparticles (Gong et al., 2021; Tran et al., 2015) or MMP-responsive hydrogels (Li et al., 2019a; Wang et al., 2019a), allows for precise drug release triggered by the unique conditions of the TME, further enhancing specificity and reducing off-target effects.

However, this approach also has several disadvantages. The invasive nature of intratumoral injection can be a concern, especially in sensitive anatomical regions of the head and neck, potentially causing pain, infection, or damage to vital structures (Harrington, 2025). The distribution of the drug within the tumor can be heterogeneous, leading to “cold spots” where cancer cells are not adequately exposed to the therapeutic agent, especially in large or irregularly shaped tumors. Tumor heterogeneity itself poses a challenge, as different regions of the tumor may respond differently to the same drug (Choi et al., 2023). Furthermore, the scalability and reproducibility of intratumoral injections in a clinical setting can be challenging, requiring specialized techniques and imaging guidance to ensure consistent delivery. Despite these challenges, the continuous innovation in biomaterial design, including active targeting ligands (e.g., RGD peptides (Javid et al., 2024; Cheng et al., 2021), folic acid (Fan et al., 2020a; Datz et al., 2016), CD44 (Chen et al., 2025), EGFR (Huang et al., 2019; Mody et al., 2021)), and stimuli-responsive mechanisms (e.g., pH, redox, enzymes, light (Liang et al., 2021; Hajebi et al., 2019; Li et al., 2020; Li et al., 2019b; Zhou et al., 2018)), continues to improve the precision and efficacy of localized drug delivery for HNC.

## 3 Mucosal or Patch-based delivery

**Fig. 2:**
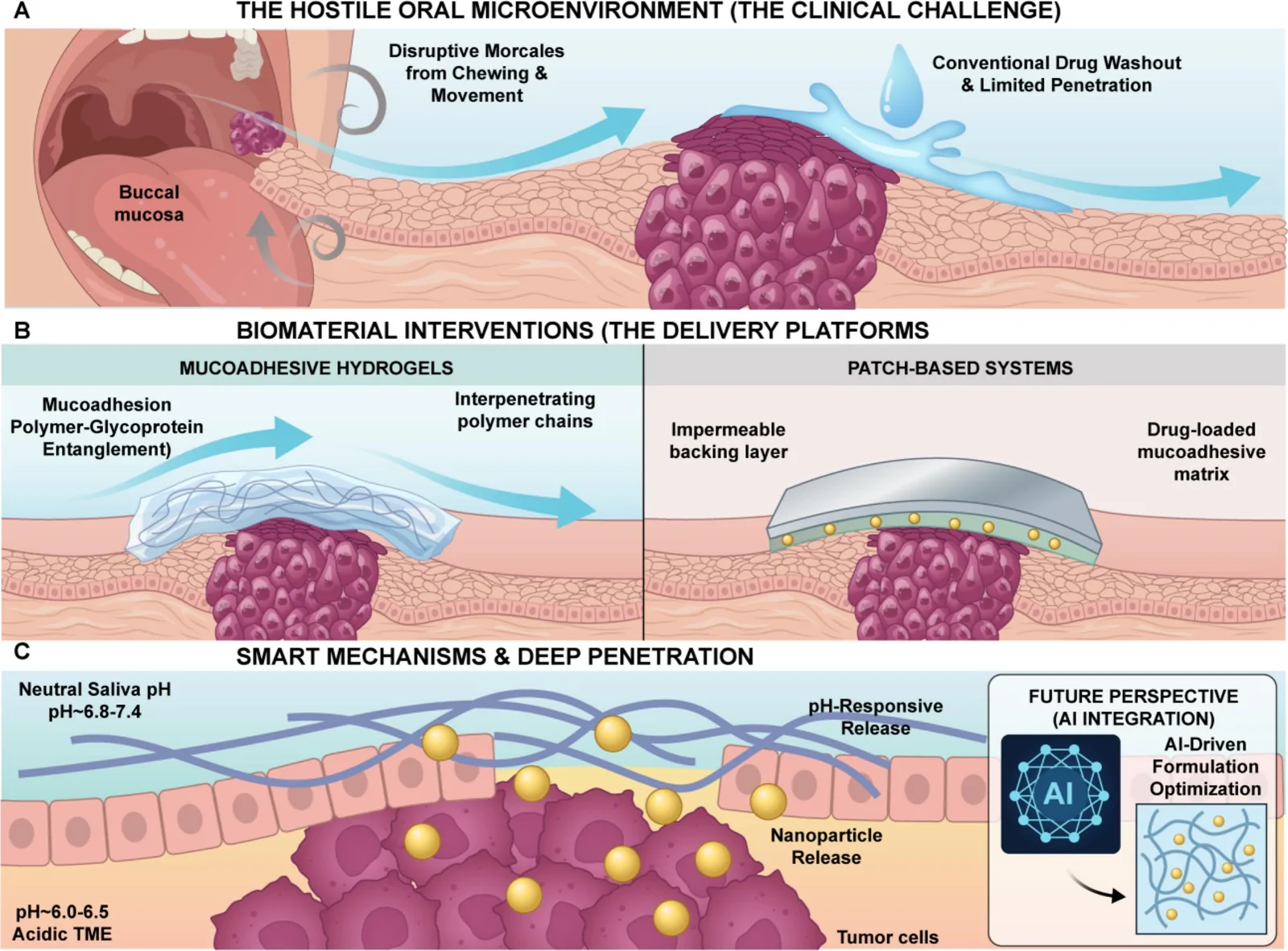
Overcoming Oral Mucosal Barriers: Biomaterial-Based Surface Delivery Systems for Head and Neck Cancer. Biomaterial-based surface delivery systems for overcoming oral mucosal barriers in head and neck squamous cell carcinoma (OSCC). (A) The clinical challenge of the hostile oral microenvironment, where the continuous flow of saliva and disruptive mechanical forces from chewing lead to the rapid washout and limited penetration of conventional topical drug formulations. (B) Biomaterial interventions to enhance local drug retention. Sub-section 1 highlights mucoadhesive hydrogels that utilize interpenetrating polymer chains to physically entangle with mucosal glycoproteins, effectively resisting salivary clearance. Sub-section 2 depicts flexible patch-based systems equipped with an impermeable backing layer and a drug-loaded mucoadhesive matrix for controlled mucosal release. (C) Smart mechanisms and deep tissue penetration. Stimuli-responsive hydrogels remain stable at the neutral pH of saliva (pH ∼6.8–7.4) but disassemble to release encapsulated nanoparticles and therapeutic payloads in the slightly acidic tumor microenvironment (pH < 6.5). Future perspectives emphasize the integration of artificial intelligence (AI) to optimize formulations and personalize these mucosal delivery platforms.

The oral cavity and pharynx are common sites for head and neck cancer, particularly OSCC. Drug delivery to these mucosal surfaces presents unique challenges due to the constant presence of saliva, which can rapidly dilute and wash away therapeutic agents, and the protective barrier function of the oral mucosa itself (Ketabat et al., 2019). Conventional topical formulations often suffer from poor retention and limited penetration, leading to suboptimal drug concentrations at the tumor site. Biomaterial-based strategies, particularly those involving mucoadhesive hydrogels and patches, offer promising solutions to overcome these barriers by enhancing local drug retention, facilitating controlled release, and improving drug penetration into the underlying tissues (Fig. 2).

### 3.1 Mucoadhesive hydrogels for oral delivery

Hydrogels are highly versatile biomaterials that can be engineered with mucoadhesive properties, allowing them to adhere to the moist mucosal surfaces of the oral cavity. This adherence prolongs the residence time of the drug delivery system, preventing rapid clearance by saliva and mechanical forces, and thereby increasing the local bioavailability of the encapsulated therapeutic agents (Wu et al., 2026; Dilixiati et al., 2026).

For instance, a multifunctional MOF-based nanohybrid (Dox/Cel/MOFs@Gel) was developed as an injectable implant platform for synergistic oral cancer therapy (Tan et al., 2020). While primarily designed as an injectable system, its gel-like nature and potential for localized application make it relevant for mucosal delivery. This nanohybrid, combining Dox and Cel, demonstrated high drug loading capacity, pH-responsive release, and potent antitumor activity against KB and SCC-9 cells in vitro. Its ability to induce tumor apoptosis and regulate angiogenesis with reduced systemic toxicity in vivo highlights its potential for local oral cancer treatment, where sustained mucosal contact could be beneficial (Tan et al., 2020).

Similarly, the development of CDDP for photodynamic therapy (PDT) to overcome cisplatin resistance in solid tumors is highly pertinent to oral cancer (Zhang et al., 2025c). The hydrogel's ability to produce singlet oxygen (^1^O_2_) rapidly and significantly decrease the IC50 for CDDP-resistant lung cancer cells under irradiation suggests its potential for localized application in OSCC, where it could adhere to the lesion and provide sustained chemo-photodynamic effects (Zhang et al., 2025c). Under continuous light irradiation, the photosensitizer ZnTAPP in the excited triplet state undergoes Type II photochemical reaction with the abundant ground-state oxygen molecules in the surrounding tissues. During this process, the photosensitizer directly transfers energy to the ground-state oxygen molecule, causing a change in its electronic spin state, thereby rapidly generating highly reactive singlet oxygen (Koh et al., 2016). Mitochondria are the main targets of singlet oxygen attacks. After the mitochondrial membrane is damaged, it will lead to the collapse of membrane potential, release pro-apoptotic factors cytochrome *c*, which binds with APAF-1 and procaspase-9 to trigger the caspase cascade reaction of Caspase-3, causing programmed cell death (Maharjan and Bhattarai, 2022).

The concept of “smart” drug delivery systems, including stimuli-responsive hydrogels, is particularly valuable for mucosal applications. These systems can be designed to release their payload in response to specific cues present in the TME, such as changes in pH, enzyme activity, or redox potential (Liang et al., 2021; Hajebi et al., 2019; Li et al., 2019b). For example, a pH-responsive hydrogel could be formulated to remain stable at the neutral pH of saliva but release drugs more rapidly in the slightly acidic environment of a tumor, ensuring targeted delivery and minimizing off-target effects.

### 3.2 Patch-based delivery Systems

Beyond injectable hydrogels, solid or semi-solid patches designed for direct application to mucosal lesions offer an alternative for localized delivery. These patches can be formulated to be mucoadhesive, flexible, and capable of controlled drug release. While specific examples of patches for HNC drug delivery are less prevalent in the provided literature compared to injectable systems, the principles of controlled release and mucoadhesion are shared.

The general advancements in “smart” delivery systems for extended drug release in cancer therapy are highly relevant, as reviewed by Kalaydina et al. (Kalaydina et al., 2018). Such systems, whether in the form of nanoparticles embedded within a patch or the patch itself being a polymeric matrix, can enhance drug delivery, bioavailability, and safety by providing sustained release at the target site. The review by Ketabat et al. on controlled drug delivery systems for oral cancer treatment specifically discusses diverse drug delivery methods and carriers, including hydrogels, and emphasizes their potential for enhancing drug bioavailability at the tumor site and reducing systemic side effects (Ketabat et al., 2019). This underscores the broader utility of localized systems, including patches, for OSCC.

### 3.3 Overcoming mucosal barriers and salivary environment

The primary advantage of mucoadhesive hydrogels and patches is their ability to significantly increase the residence time of therapeutic agents on the mucosal surface. This prolonged contact allows for greater drug absorption into the tumor tissue and reduces the frequency of administration, improving patient compliance. The controlled release kinetics offered by these biomaterials ensure a steady therapeutic concentration over an extended period, which is crucial for maximizing efficacy against slow-growing or recurrent tumors.

However, several challenges remain. The mechanical forces within the oral cavity, such as chewing and tongue movement, can dislodge patches or disrupt hydrogel integrity, limiting their effective residence time. The taste and texture of oral formulations can also impact patient acceptance and compliance. Furthermore, achieving adequate drug penetration through the stratified squamous epithelium of the oral mucosa, especially into deeper tumor tissues, can still be challenging. The thickness and integrity of the mucosal barrier can vary significantly depending on the location and the patient's condition (e.g., mucositis induced by prior treatments).

To address these limitations, future research could focus on developing biomaterials with stronger mucoadhesive properties, improved mechanical strength, and enhanced permeability enhancers. Incorporating nanoparticles within hydrogel matrices or patches could further improve drug penetration and cellular uptake, as seen with the Er/Cm-NP-HG system for intratumoral delivery (Haider et al., 2024). Additionally, stimuli-responsive systems that can adapt to the dynamic oral environment or respond to specific tumor cues could offer more intelligent and effective mucosal delivery strategies. The integration of artificial intelligence in biomaterial design, as discussed by Miao et al. (Miao et al., 2026), could also play a role in optimizing the formulation and personalization of mucosal delivery systems for oral oncology, improving diagnostic accuracy and guiding drug carriers and dosing.

In summary, while mucosal and patch-based delivery for HNC is still largely in preclinical stages, the inherent advantages of localized, sustained release, and reduced systemic toxicity make it a highly promising area. Continued innovation in biomaterial science, focusing on mucoadhesion, controlled release, and tissue penetration, is essential to translate these strategies into effective clinical treatments for oral and pharyngeal cancers.

## 4 Postoperative localized controlled-release systems

**Fig. 3:**
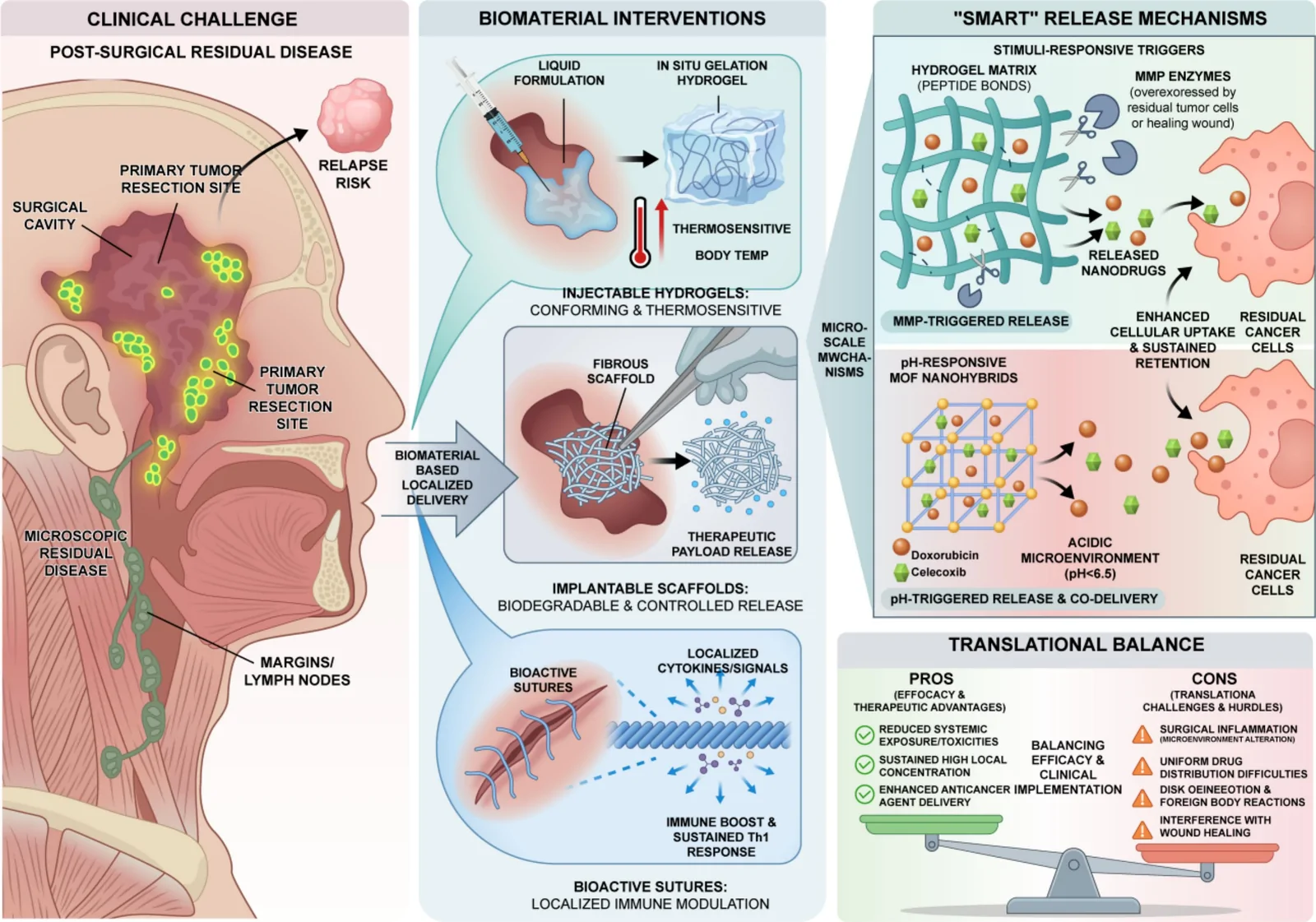
Postoperative Biomaterial Interventions: Eradicating Residual Disease in Head and Neck Cancer. Biomaterial-mediated localized delivery strategies for post-surgical tumor management and the eradication of residual disease. (Panel A) The clinical challenge following primary tumor resection, highlighting the high risk of disease relapse due to microscopic residual cancer cells remaining at the surgical margins or in regional lymph nodes. (Panel B) Meso-scale biomaterial interventions designed to conform to complex surgical cavities. These include thermosensitive injectable hydrogels (e.g., chitosan) that undergo in situ gelation at body temperature, implantable biodegradable fibrous scaffolds for controlled therapeutic payload release, and bioactive sutures that provide localized cytokines to boost a sustained Th1 immune response. (Panel C) Micro-scale “smart” release mechanisms triggered by the local microenvironment. Hydrogel matrices can be degraded by matrix metalloproteinases (MMPs) overexpressed by residual tumor cells or the healing wound, leading to localized nanodrug release. Additionally, pH-responsive metal-organic framework (MOF) nanohybrids co-deliver therapeutic agents like Doxorubicin (Dox) and Celecoxib (Cel) under acidic conditions. (Panel D) The translational balance of postoperative systems, weighing the therapeutic advantages of reduced systemic toxicity and high local drug concentration against translational hurdles such as surgical inflammation, uniform drug distribution, and wound healing interference.

Local recurrence after surgical resection remains a major clinical challenge in HNC, significantly impacting patient survival and quality of life (Johnson et al., 2020). Despite radical surgery and adjuvant therapies like radiotherapy or chemoradiotherapy, microscopic residual disease at the surgical margins or in regional lymph nodes often leads to disease relapse. The goal of postoperative localized controlled-release systems is to deliver therapeutic agents directly to the surgical bed or surrounding tissues over an extended period, thereby eradicating residual cancer cells, preventing recurrence, and potentially reducing the need for or intensity of systemic adjuvant treatments. Biomaterials are uniquely suited for this application, offering sustained drug release, biocompatibility, and the ability to conform to complex surgical cavities (Fig. 3).

### 4.1 Injectable hydrogels for postoperative drug delivery

Injectable hydrogels are particularly attractive for postoperative applications because they can be easily administered into irregular surgical defects, where they solidify or gel in situ to form a drug-releasing depot. This allows for intimate contact with the residual tumor cells and surrounding tissues, ensuring high local drug concentrations.

A prime example is the multifunctional MOF-based nanohybrid (Dox/Cel/MOFs@Gel) designed as an injectable implant platform for synergistic oral cancer therapy (Tan et al., 2020). This system, combining Dox and Cel, demonstrated high drug loading capacity and pH-responsive release. Crucially, in vivo studies showed that this nanohybrid induced tumor apoptosis and regulated angiogenesis with reduced systemic toxicity, making it a promising candidate for local oral cancer treatment. Such a system could be injected into the surgical cavity after tumor removal, providing sustained release of both chemotherapeutic and anti-inflammatory agents to target any remaining cancer cells and modulate the local microenvironment to prevent recurrence. The sustained release profile is critical here, as it ensures therapeutic levels are maintained over weeks or months, addressing the challenge of microscopic residual disease.

Similarly, the nano dox-indocyanine green (ICG) MMP-responsive hydrogel (NDIMH) developed for chemophototherapy in HNSCC could be adapted for postoperative use (Wang et al., 2019a). Its MMP-2-responsive release mechanism means that drug release would be triggered by enzymes often overexpressed in residual tumor cells or the healing wound microenvironment. The improved nanodrug retention at the tumor site observed in vivo suggests that such a hydrogel could effectively “coat” the surgical bed, providing a localized and sustained therapeutic effect. Another MMP-responsive HA hydrogel loaded with doxorubicin-encapsulated micelles also demonstrated local sustained DOX release and tumor growth inhibition without organ damage in vivo (Li et al., 2019a). This system's ability to degrade faster in tumors, combined with its sustained release, makes it an excellent candidate for preventing local recurrence by targeting residual disease.

The thermosensitive chitosan hydrogels incorporating Er and Cm-loaded nanoparticles (optEr/Cm-NP-HG) also show potential (Haider et al., 2024). These hydrogels, which exhibit sustained drug release and enhanced cytotoxicity against HNSCC cells, could be injected as a liquid and then gel at body temperature within the surgical site, providing a localized and prolonged therapeutic effect. The combination of targeted nanoparticles within a hydrogel matrix offers a dual advantage: the nanoparticles provide enhanced cellular uptake and targeting, while the hydrogel ensures sustained local retention.

### 4.2 Implantable devices and bioactive scaffolds

Beyond injectable hydrogels, solid or semi-solid implantable devices and bioactive scaffolds can be surgically placed into the resection cavity. These systems can be designed to degrade slowly over time, releasing their therapeutic payload.

While not explicitly a drug delivery system, the development of a TRACER 3D co-culture tumor model for head and neck cancer using fibrous scaffolds highlights the potential for biomaterial scaffolds to mimic the TME and study tumor-stroma interactions (Young et al., 2018). This research, though focused on modeling, informs the design of scaffolds that could potentially be loaded with drugs and implanted postoperatively.

The concept of bioactive sutures, which induced a sustained Th1 immune response in vitro, could also be considered for postoperative applications (Shibuya et al., 2004). Sutures are routinely used in surgical closures, and if functionalized with immunomodulatory agents, they could provide a localized immune boost in the surgical bed, potentially clearing microscopic disease and preventing recurrence. This represents a novel in situ strategy for enhancing the immune system directly at the site of resection.

### 4.3 Advantages and disadvantages of postoperative systems

The primary advantage of postoperative localized controlled-release systems is their ability to deliver a sustained, high concentration of anticancer agents directly to the site of highest recurrence risk, while significantly reducing systemic exposure and associated toxicities. This approach can potentially improve local control rates, reduce the need for or intensity of conventional adjuvant therapies, and enhance patient quality of life. The ability of biomaterials to conform to complex surgical geometries and provide tunable release kinetics further enhances their utility. For instance, the Dox/Cel/MOFs@Gel system demonstrated reduced systemic toxicity while achieving potent antitumor activity (Tan et al., 2020).

However, there are several challenges associated with these systems. The surgical procedure itself introduces inflammation and alters the tissue microenvironment, which can affect the stability, degradation, and drug release profile of the implanted biomaterial. Ensuring uniform distribution of the drug throughout the entire surgical bed, especially in areas with microscopic disease, can be difficult. The biocompatibility and biodegradability of the biomaterial are paramount; the implant must not cause excessive inflammation, foreign body reactions, or interfere with wound healing. The risk of infection at the surgical site, particularly with foreign materials, must also be carefully managed. Furthermore, the manufacturing and sterilization of such implantable systems need to meet stringent regulatory standards.

Another critical consideration is the timing and duration of drug release. The ideal release profile needs to be carefully tailored to the specific cancer type, stage, and individual patient characteristics. An excessively rapid release rate may induce local toxicity, whereas an overly sluggish release profile could prove ineffective against rapidly proliferating residual cells. The integration of “smart” stimuli-responsive features, such as those responsive to tumor-specific enzymes or pH, could help optimize drug release in response to the presence of residual cancer cells (Liang et al., 2021; Li et al., 2019b).

The current body of evidence for these systems in HNC is predominantly preclinical, with most studies demonstrating efficacy in animal models or in vitro settings. Translating these findings to the clinic requires rigorous evaluation of safety, efficacy, and optimal dosing strategies in human patients. The complexity of HNC tumor heterogeneity and the variability in surgical outcomes further complicate the design of clinical trials for such localized systems. Nevertheless, the potential benefits in preventing local recurrence and improving patient outcomes make postoperative localized controlled-release systems a highly promising area of biomaterial research for HNC.

## 5 Combinations with radiotherapy, chemotherapy, immunotherapy, and photodynamic therapy

The complex and aggressive nature of HNC, coupled with its high rates of local recurrence and metastasis, often necessitates multimodal therapeutic approaches. Combination therapies, integrating conventional treatments like radiotherapy and chemotherapy with newer modalities such as immunotherapy and PDT, aim to achieve synergistic effects, overcome drug resistance, and improve patient outcomes (Johnson et al., 2020; Liang et al., 2021). Biomaterial-based drug delivery systems are uniquely positioned to facilitate these complex combinations by enabling the precise spatiotemporal delivery of multiple agents, sensitizing tumors to other treatments, and modulating the TME to enhance therapeutic responses.

### 5.1 Combination with chemotherapy

Chemotherapy remains a cornerstone of HNC treatment, but its efficacy is often limited by systemic toxicity, poor tumor accumulation, and the development of drug resistance (Mohit et al., 2026; Han et al., 2024). Biomaterials can significantly enhance chemotherapy by improving drug delivery and overcoming resistance mechanisms.

Many nanoparticle systems are designed to deliver chemotherapeutic agents more effectively. For example, redox-responsive micelles based on HSDs (hydrophilic-hydrophobic-hydrophilic triblock copolymers) were developed for laryngopharyngeal carcinoma therapy, achieving high DOX loading and near 100% DOX release in the presence of high intracellular GSH concentrations, demonstrating enhanced cellular uptake and selectivity against cancer cells (Sun et al., 2018). This approach reduces systemic toxicity while maximizing local drug effect. Similarly, paclitaxel-loaded ROS-responsive nanoparticles (PTX@TPGS-PBTE NPs) showed enhanced cytotoxicity and apoptosis in SCC-7 cells and significant tumor growth inhibition in murine models, especially with cRGD modification for targeted delivery (Tu et al., 2023). The use of d-α-tocopheryl polyethylene glycol succinate (TPGS) in such formulations is notable, as TPGS enhances drug solubility, permeation, and has antitumor activity, including inhibiting P-glycoprotein to combat multi-drug resistance (MDR) (Yang et al., 2018; Tan et al., 2017).

Biomimetic nanoplatforms also excel in chemo-delivery. P-T-p@CM nanoparticles, for instance, were developed for dual-pathway metabolic blockade in HNSCC, delivering telaglenastat (GLS1 inhibitor) and CRISPR-Cas9 plasmid targeting HIF-1α (Fang et al., 2026). This system achieved precise tumor homing and stimuli-responsive release, leading to significant metabolic reduction and a 90% tumor inhibition rate in vivo, demonstrating the power of co-delivery for synergistic chemosensitization (Fang et al., 2026). Another example is PPPt^IV NPs, which co-encapsulated a Pt^IV prodrug and CRISPR/Cas9-PKM2 plasmids (Zou et al., 2025). These nanoparticles induced DNA damage, ROS, and apoptosis, while reducing lactate and HIF-1α/PD-L1, showing superior efficacy against HNSCC tumors compared to cisplatin with reduced nephrotoxicity, highlighting the potential for chemo-gene co-delivery.

Hydrogels also play a role in combination chemotherapy. The multifunctional MOF-based nanohybrid (Dox/Cel/MOFs@Gel) combined Dox and celecoxib for synergistic oral cancer therapy, showing potent antitumor activity and reduced systemic toxicity (Tan et al., 2020). This injectable platform allows for sustained co-delivery, which is crucial for maintaining therapeutic concentrations over time. CDDP was developed to overcome cisplatin resistance, significantly decreasing the IC50 for CDDP-resistant lung cancer cells under irradiation, suggesting its potential for chemo-photodynamic combination in HNC (Zhang et al., 2025c).

The combination of cisplatin with other agents is a common strategy. For p53-mutated head and neck cancers, combination treatment with cisplatin and PG3 showed synergistic effects, with PG3 enhancing cisplatin sensitivity and reducing side effects by lowering the cisplatin dose (Tian and El-Deiry, 2025). While not explicitly biomaterial-based, this highlights a therapeutic strategy that could be enhanced by biomaterial delivery. Similarly, p90RSK pathway inhibition synergizes with cisplatin in TMEM16A overexpressing HNC, overcoming cisplatin resistance, a mechanism that could be targeted by biomaterial-delivered inhibitors (Yassin-Kassab et al., 2024).

### 5.2 Combination with radiotherapy

Radiotherapy is a primary treatment modality for HNC, but radioresistance and damage to surrounding healthy tissues remain significant challenges (Johnson et al., 2020). Biomaterials can act as radiosensitizers, enhance radiation delivery, or protect normal tissues.

AuNPs are well-known radiosensitizers due to their high atomic number, which enhances local dose deposition under radiation. While not explicitly detailed in the provided abstracts for HNC radiosensitization, the general principle of AuNPs in PTT and imaging suggests their potential for enhancing radiotherapy (Hang et al., 2024).

More directly, FTO inhibition has been shown to enhance RT efficacy in HPV-negative HNSCC tumor xenografts (Ji et al., 2025). Genetic and pharmacologic FTO inhibition radiosensitized cells by amplifying DNA damage and reducing RAD51 foci formation without increasing oral mucositis, suggesting FTO inhibition as a strategy to improve RT therapeutic index. Biomaterial-based delivery of FTO inhibitors could provide localized radiosensitization.

Gold-siRNA supraclusters (BSCgal) have been shown to augment the radiodynamic and immunotherapeutic effects of stereotactic ablative radiotherapy (SABR) at primary and metastatic tumors in mice models of HNC (Jiang et al., 2025). This was achieved by promoting tumor-inhibitory leukocytes, upregulating cytotoxic granzyme B, and reducing immunosuppressive cell populations, demonstrating a powerful combination of nanotechnology, gene therapy, and radiotherapy.

The use of bio-printed organoids in HNSCC for functional precision medicine and radiotherapy investigation is also emerging (Lin et al., 2024). This platform allows for high-throughput ex vivo drug screening to investigate radiotherapy and chemotherapy interactions, which can guide the development of biomaterial-based radiosensitizers and combination regimens.

### 5.3 Combination with immunotherapy

Immunotherapy, particularly immune checkpoint inhibitors (ICIs), has revolutionized cancer treatment, but response rates in HNC are variable, and many tumors remain “cold” or immune-excluded (Johnson et al., 2020; Wu et al., 2024b). Biomaterials can reprogram the TME, enhance immune cell infiltration, and improve the efficacy of immunotherapeutics.

Nanomedicine offers a promising strategy for enhancing tumor immunotherapy efficacy in HNSCC (Li et al., 2024a). Nanoparticles can overcome biological barriers and improve drug delivery efficiency, thereby enhancing the effects of ICIs. For instance, cell-inspired biomaterials are being tailored to fuel cancer therapy, offering high efficacy, targeting, and spatiotemporal control, which are crucial for modulating immune responses (Wang et al., 2025).

Metabolic reprogramming by chemo-gene co-delivery nanoparticles (PPPt IV NPs) has been shown to activate T cell-mediated antitumor immunity in HNSCC (Zou et al., 2025). By co-encapsulating a Pt IV prodrug and CRISPR/Cas9-PKM2 plasmids, these NPs reduced lactate and HIF-1α/PD-L1, enhanced dendritic cell maturation, M1 macrophage polarization, and altered cytokine profiles, leading to superior efficacy against HNSCC tumors. This highlights a sophisticated approach to combine chemotherapy with immune activation.

Zinc-mediated metalloimmunotherapy with dual elimination of tumor and intratumoral bacteria in OSCC is another innovative strategy (Zhou et al., 2025). PYT@ZIF8siRNA NPs, loaded with pyrithione and siRNA for SLC30A1, caused zinc overload, leading to OSCC cell death and *P. gingivalis* elimination. These NPs also stimulated antitumor immunity and hindered epithelial malignant transformation. Combined with PD-1 blockade, they reprogrammed the TME, suggesting a new approach for precision metalloimmunotherapy in OSCC (Zhou et al., 2025).

Nanosized shikonin-loaded, CGKRK-modified lipid nanoparticles (C-SNPs) induced necroptosis in HNSCC and suppressed mismatch repair, activating cGAS-mediated IFN response and PD-L1 expression (Yu et al., 2025). C-SNPs combined with anti-PD-1 antibody increased DC and CD8+ T cell infiltration. Further enhancement with Mn2 + −modified C-SMNPs activated cGAS-STING signaling, leading to sustained tumor suppression in vivo, demonstrating therapeutic potential for HNSCC immunotherapy (Yu et al., 2025). Manganese-derived biomaterials, in general, are being explored for their ability to regulate the TME and enhance immunotherapy (Huang et al., 2024).

Hydrogels can also modulate the immune response. A self-assembling RADA16-I peptide in situ hydrogel loaded with Celastrol (RADA-CeT) boosted ICD in OSCC, exhibiting excellent anti-tumor efficacy by amplifying ICD, which is critical for initiating an effective immune response (Zhang et al., 2025b). Mechanistic studies of hydrogels in oral cancer treatment highlight their synergistic effects of drug delivery and immune modulation, enabling targeted, stimuli-responsive drug release and enhancing local immune responses (Dilixiati et al., 2026).

Clinical trials are already exploring biomaterial-related immunotherapy strategies. An anchored IL-12 drug conjugate (ANK-101) showed promising results in a Phase I trial for advanced solid tumors, inducing local PD-L1 expression, CD8+ T cell recruitment, and immune activation (Park et al., 2025). While not explicitly a biomaterial delivery system in the abstract, the concept of an “anchored drug conjugate” implies a localized delivery mechanism. Similarly, a Phase 3 randomized study of ASP-1929 photoimmunotherapy (PIT) in combination with pembrolizumab versus standard of care in locoregional recurrent HNSCC (LR rHNSCC) aims to improve survival (Maniakas et al., 2025). PIT, which uses a photosensitizer conjugated to an antibody, is a form of targeted phototherapy that can also induce immune responses. The combination with pembrolizumab (an ICI) leverages the potential for PIT to convert “cold” tumors into “hot” ones, enhancing the efficacy of immunotherapy (Allen et al., 2025).

### 5.4 Combination with photodynamic and photothermal therapy

PDT and PTT are light-activated therapies that offer localized, non-invasive treatment options. Biomaterials, particularly nanoparticles, are crucial for enhancing the delivery and efficacy of photosensitizers and photothermal agents, overcoming their limitations such as poor solubility, aggregation, and lack of tumor specificity (Son et al., 2019; Fan et al., 2020b).

Porphyrin nanoparticles (Porphysomes) have been shown to enhance fluorescence and photoacoustic imaging of head and neck tumors, enabling complete targeted ablation of buccal and tongue carcinomas (Muhanna et al., 2015). Porphysome-enabled PTT demonstrated superiority over surgery in eradicating primary tumors and metastatic lymph nodes while preserving adjacent structures, highlighting its potential for precise tumor diagnosis and treatment (Muhanna et al., 2015). Similarly, pyropheophorbide-a-loaded polyethylene glycol–poly(lactic-*co*-glycolic acid) nanoparticles (NPs) were developed for tumor-targeted PDT, showing high tumor site enrichment and ablating tumors in a xenograft KB tumor mouse model (Liu et al., 2016).

AuNPs are widely used for PTT due to their efficient conversion of light into heat (Hang et al., 2024). A multifunctional gold nanoplatform (PDPN Ab- and DOX-conjugated AuNPs) was designed for combined chemo-PTT against oral cancer, demonstrating enhanced antitumor efficacy in vitro and in vivo (Liu et al., 2020). Core-satellite mesoporous silica‑gold nanocomposites (MSN–Fe–AuNPs) also exhibited pH-dependent photothermal properties and H_2_O_2_-induced radical generation, leading to complete tumor inhibition after combined chemo-photothermal treatment in vivo (Jin et al., 2018). Polydopamine (PDA) nanomaterials, due to their unique properties, are also being explored in PTT and combination therapies for HNC (Witkowska et al., 2023). PDA-coated AuNPs, for instance, exhibit high photothermal stability and efficiency, and a single PTT session with sub-therapeutic doxorubicin eliminated local and distant tumors in animal models, revealing an immunological aspect of chemo- PTT (Nam et al., 2018).

Stimuli-responsive systems are particularly advantageous for PDT/PTT. Charge-reversal biodegradable MSNs exhibited photothermal properties and enhanced chemo/ PTT with MRI contrast for monitoring (Li et al., 2021). Chitosan capped pH-responsive hollow MSNs (HMSN-GM-CS-FA) co-delivered PA and DOX, combining photothermal, photodynamic, and chemotherapies for synergistic antitumor efficacy (Yan et al., 2020). A near-infrared light-triggered human serum albumin drug delivery system with coordination bonding of ICG and CDDP (HSA-ICG-DDP NPs) was developed for targeted photochemistry therapy against OSCC (Wang et al., 2019b). This system showed synergistic effects with PDT, PTT, and chemotherapy, enhancing therapeutic efficacy by leveraging SPARC expression in OSCC and CAFs for cellular uptake, and NIR-induced photothermal effect to trigger DDP release and ROS generation (Wang et al., 2019b). SPARC activates the TGF-β/Smad pathway in CAFs and further secretes factors such as IL-6 and CXCL12 to construct an immunosuppressive microenvironment for tumor cell invasion (Li et al., 2024b). Multiple studies have confirmed that the high expression of SPARC in OSCC tissues is significantly positively correlated with advanced TNM stage, positive lymph node metastasis and poor prognosis of patients, making it an ideal therapeutic target and biomarker (Jing et al., 2019). SPARC naturally has a high affinity for collagen and basement membrane components which is useful for designing nanosystems. The surface of nanocarriers is modified with collagen-mimics peptides or the binding domain of SPARC itself by the matrix network rich in SPARC-collagen complexes when passing through the tumor stroma, thereby prolonging the local retention time (Gaber et al., 2019). Similarly, ICG-containing polyglutamate (PGA) nanoparticles, which are cathepsin B-degradable and NIR-responsive, showed enhanced cytotoxicity when combined with saporin due to photo-induced release, demonstrating potential for treating superficial malignancies like head and neck tumors (Tarassoli et al., 2017). ROS-responsive nanoparticles based on PEGylated prodrug were designed for targeted treatment of oral tongue squamous cell carcinoma (OTSCC) by combining PDT and chemotherapy, showing tumor targeting and growth suppression after local laser irradiation (Shi et al., 2018).

### 5.5 Advantages and disadvantages of combination therapies with biomaterials

The primary advantage of using biomaterials in combination therapies for HNC is the ability to achieve synergistic effects, overcome drug resistance, and reduce systemic toxicity. Biomaterials allow for the co-delivery of multiple agents with precise spatiotemporal control, ensuring that drugs are released at the right time and place to maximize their combined therapeutic impact. For example, the metabolic reprogramming achieved by PPPt^IV NPs (Zou et al., 2025) and the immune activation by C-SNPs (Yu et al., 2025) demonstrate how biomaterials can fundamentally alter the tumor's response to therapy. The ability to combine imaging with therapy (theranostics), as seen with porphyrin nanoparticles^87^ and SeNPs (Huang et al., 2019), further enhances treatment precision.

However, the complexity of designing and manufacturing multifunctional biomaterials for combination therapy introduces several challenges. Ensuring the stability and controlled release of multiple drugs with different physicochemical properties can be difficult. The potential for drug-drug interactions within the delivery system or in the biological environment must be carefully evaluated. The optimal dosing and timing of each component in a combination regimen, especially when one component is light-activated or immune-modulating, requires extensive preclinical optimization. Furthermore, the regulatory pathway for approving complex combination products involving novel biomaterials and multiple active pharmaceutical ingredients can be lengthy and challenging.

Despite these complexities, the continuous advancements in biomaterial science, coupled with a deeper understanding of HNC biology and resistance mechanisms, are paving the way for more effective and personalized combination therapies. The integration of artificial intelligence in biomaterial design and optimization, as highlighted by Miao et al. (Miao et al., 2026), could further accelerate the development of these sophisticated systems, leading to more precise, efficient, and tailored solutions for HNC management.

## 6 Translation challenges

**Fig. 4:**
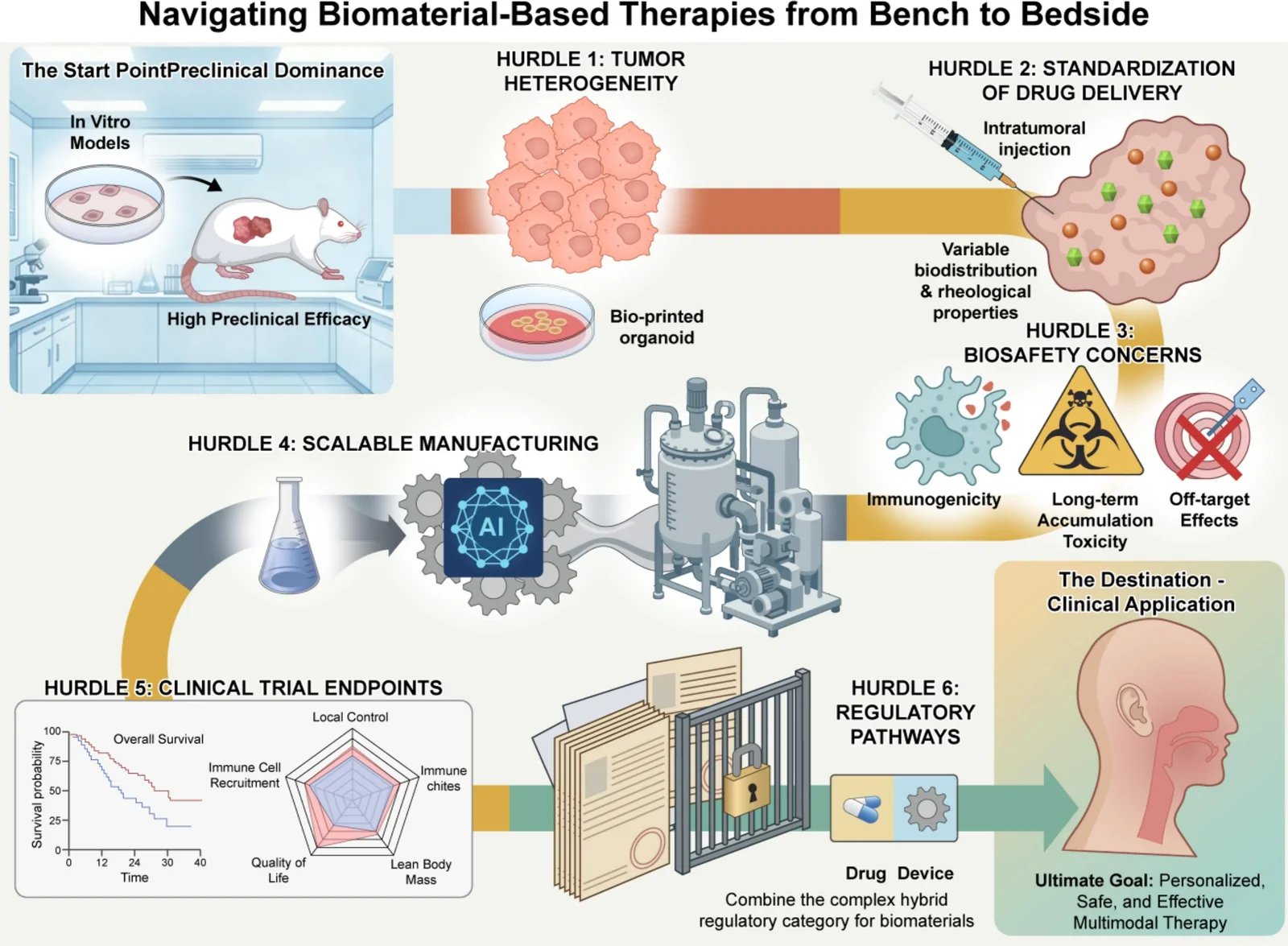
Navigating the Translational Chasm: From Preclinical Promise to Clinical Application in Head and Neck Cancer. The translational pathway and primary challenges in advancing biomaterial-based therapies from bench to bedside for head and neck cancer (HNC). The starting point reflects the current predominance of preclinical evidence, where high efficacy is frequently observed under highly controlled in vitro conditions and in in vivo animal models. (Center) The winding translational pathway is obstructed by six major clinical hurdles. Hurdle 1 highlights tumor heterogeneity, necessitating functional precision medicine and patient stratification via bio-printed organoids and gene signatures. Hurdle 2 emphasizes the lack of standardization in drug delivery, particularly the variable biodistribution and rheological properties encountered during intratumoral injections. Hurdle 3 encompasses biosafety concerns, including immunogenicity, long-term accumulation, toxicity, and off-target effects. Hurdle 4 depicts the bottleneck of scalable manufacturing, where artificial intelligence (AI) may assist in streamlining the transition from laboratory batches to industrial-scale production. Hurdle 5 represents the complexity of designing appropriate clinical trial endpoints, balancing overall survival (OS) with surrogate markers like local control, immune cell recruitment, and quality of life. Hurdle 6 illustrates the complex regulatory pathways required for hybrid drug-device biomaterial products. The ultimate clinical destination envisions personalized, safe, and effective multimodal therapy successfully implemented in human patients.

While biomaterial-based drug delivery strategies hold immense promise for revolutionizing HNC treatment, their translation from preclinical research to widespread clinical application is fraught with significant challenges. It is crucial to rigorously distinguish between evidence derived from animal and in vitro studies versus early-stage clinical explorations, as the leap from controlled laboratory settings to the complex human physiological environment is substantial. The current body of evidence, as reflected in the provided literature, remains predominantly focused on HNSCC and OSCC, with a strong emphasis on in vitro and in vivo animal models, and only a few examples of early-stage clinical trials or concepts for human application. Clinical translatability is still constrained by a multitude of factors, necessitating a cautious yet optimistic outlook (Fig. 4).

### 6.1 Predominance of preclinical evidence

A review of the provided literature clearly indicates that the vast majority of studies on biomaterial-based drug delivery for HNC are conducted at the preclinical stage. Researchers are actively developing and characterizing novel nanoparticles, hydrogels, and other systems in cell culture models and xenograft mouse models. For instance, the biomimetic nanoplatform P-T-p@CM achieved a 90% tumor inhibition rate in vivo after 15 days (Fang et al., 2026), and PPPt^IV NPs showed superior efficacy against HNSCC tumors in mouse models (Zou et al., 2025). Er and Cm-loaded nanoparticles embedded in chitosan hydrogels demonstrated tumor growth inhibition in HNSCC mouse models (Haider et al., 2024). Porphyrin nanoparticles enabled complete targeted ablation of buccal and tongue carcinomas in rabbit and hamster models (Muhanna et al., 2015). LNPIT delivering mRNA led to anti-tumor responses in mice (Huayamares et al., 2025). Zein nanoparticles loaded with an anticancer peptide reduced IC50 values and increased apoptosis in HNSCC cell lines (Jenča Jr et al., 2026). Targeted CRISPR-LNP delivery inhibited tumor growth by 90% and increased survival in xenograft mouse models (Masarwy et al., 2025). Hypoxia-targeting multifunctional nanoparticles effectively destroyed tumors in vivo with reduced systemic toxicity (Song et al., 2020). The MMP-responsive hydrogel showed favorable synergistic antitumor efficacy and biosafety in vivo (Wang et al., 2019a), and doxorubicin-loaded HA hydrogels inhibited tumor growth without organ damage in vivo (Li et al., 2019a). BSCgal augmented radiodynamic and immunotherapeutic effects in mouse models (Jiang et al., 2025). Nanosized shikonin-loaded lipid nanoparticles led to sustained tumor suppression in vivo (Yu et al., 2025). 177Lu-labeled polymeric nanoparticles suppressed tumor growth and extended survival in mice (Hsieh et al., 2025). Paclitaxel-loaded ROS-responsive nanoparticles inhibited tumor growth in murine models (Tu et al., 2023). The multifunctional MOF-based nanohybrid induced tumor apoptosis and regulated angiogenesis in vivo (Tan et al., 2020). Glutathione-sensitive and folate-targeted nanoparticles showed effective antitumor effects in OSCC murine models (Fan et al., 2020a). The gold nanoplatform demonstrated enhanced antitumor efficacy in vitro and in vivo (Liu et al., 2020). The Pt-loaded nanocomposite exhibited strong inhibitory effects on tumor growth in OSCC xenograft mice (Wei et al., 2018). ROS-responsive nanoparticles suppressed tumor growth in tumor-bearing mice (Shi et al., 2018).

These studies, while demonstrating compelling proof-of-concept and significant therapeutic potential, are conducted under highly controlled experimental conditions that often do not fully recapitulate the complexity of human HNC. Factors such as tumor size, immune system competence, genetic heterogeneity, and the presence of comorbidities in human patients are often simplified or absent in animal models. The transition from these promising preclinical results to successful clinical trials requires overcoming substantial hurdles.

### 6.2 Constraints on clinical translatability

#### 6.2.1 Tumor heterogeneity

HNC is characterized by significant intratumoral and intertumoral heterogeneity, both genetically and phenotypically (Johnson et al., 2020; Choi et al., 2023). This means that different regions within the same tumor, or tumors from different patients, can exhibit varying molecular profiles, cellular compositions, and microenvironmental characteristics. This heterogeneity poses a major challenge for targeted drug delivery systems, as a biomaterial designed to target a specific receptor or respond to a particular TME cue might only be effective in a subset of cancer cells or patients. For example, while CD44-targeted nanomedicine shows promise for HNSCC by eliminating BMI1+ CSCs (Chen et al., 2025), the expression of CD44 and BMI1 can vary. Similarly, the efficacy of EGFR-targeted therapies, which could be delivered by biomaterials, is predicted by a 13-gene signature, highlighting the need for patient stratification (Liao et al., 2026). The development of personalized medicine approaches, guided by biomarkers and functional precision medicine platforms like bio-printed organoids, is crucial to address this heterogeneity (Lin et al., 2024; Champlin et al., 2025; Okuyama et al., 2026).

#### 6.2.2 Standardization of drug delivery

Achieving standardized and reproducible drug delivery in a clinical setting is critical for consistent therapeutic outcomes. For localized delivery methods, such as intratumoral injections, ensuring that the biomaterial is uniformly distributed throughout the tumor or surgical bed can be challenging, especially in irregularly shaped tumors or those in anatomically complex regions (Huang et al., 2020; Harrington, 2025). Factors like injection volume, rate, needle gauge, and the rheological properties of the biomaterial can all influence distribution and retention. For systemic delivery of nanoparticles, controlling their biodistribution, accumulation in the tumor, and cellular uptake can be variable among patients due to differences in tumor vascularization, lymphatic drainage, and individual physiological responses. The lack of standardized protocols for administration and monitoring of biomaterial-based therapies hinders their widespread adoption.

#### 6.2.3 Biosafety concerns

The biocompatibility, biodegradability, and long-term safety of biomaterials are paramount for clinical translation. While many preclinical studies report good biocompatibility, the long-term effects of novel materials, especially nanoparticles, in humans are not fully understood (Ma et al., 2024). Potential concerns include: (1) **Immunogenicity:** Biomaterials, particularly those derived from natural sources or with complex surface modifications, could elicit undesirable immune responses, leading to inflammation or rapid clearance. (2) **Toxicity:** Degradation products of biodegradable biomaterials must be non-toxic and easily cleared from the body. Non-biodegradable materials raise concerns about long-term accumulation and potential chronic toxicity. For example, while AuNPs are generally considered safe, their long-term fate and potential for accumulation in organs need thorough investigation (Hang et al., 2024). (3) **Off-target effects:** Despite efforts to achieve targeted delivery, some degree of off-target accumulation is often unavoidable, potentially leading to adverse effects in healthy tissues. (4) **Manufacturing impurities:** The synthesis of complex biomaterials can introduce impurities that may have toxic effects. The rigorous evaluation of these biosafety aspects in large animal models and eventually in human clinical trials is a lengthy and expensive process.

#### 6.2.4 Scalable manufacturing processes

Translating biomaterial-based therapies from laboratory bench to industrial scale requires robust, reproducible, and cost-effective manufacturing processes. Many advanced biomaterial formulations, especially complex nanoparticles or stimuli-responsive hydrogels, are currently produced in small batches using specialized laboratory techniques. Scaling up these processes while maintaining consistent quality, particle size, drug loading, and release kinetics is a significant hurdle. Issues such as batch-to-batch variability, sterilization methods, and stability during storage and transport need to be addressed. The economic feasibility of producing these advanced therapies at a scale sufficient for widespread clinical use is also a major consideration. Artificial intelligence in biomaterial design and optimization could potentially aid in streamlining manufacturing processes and quality control (Miao et al., 2026).

#### 6.2.5 Appropriate endpoint designs in clinical trials

Designing clinical trials for novel biomaterial-based drug delivery systems in HNC requires careful consideration of appropriate endpoints. Beyond traditional measures like overall survival (OS) and progression-free survival (PFS), surrogate endpoints that reflect local drug activity, tumor response, and reduction in treatment-related toxicity are crucial. For localized therapies, endpoints related to local control, surgical margin status, and prevention of local recurrence are highly relevant. For combination therapies, demonstrating synergistic effects and improved therapeutic index (efficacy vs. toxicity) is key. The use of advanced imaging techniques and molecular biomarkers to assess drug delivery, target engagement, and biological response in real-time will be essential. For example, the Phase I trial of anchored IL-12 drug conjugate (ANK-101) measured local PD-L1 expression and CD8+ T cell recruitment as indicators of biologic activity (Park et al., 2025). The Phase 3 study of ASP-1929 photoimmunotherapy combined with pembrolizumab uses OS as a primary endpoint, but also complete response rate (CRR) and objective response rate (ORR) as secondary endpoints (Maniakas et al., 2025). The Phase II trial of nano-crystalline megestrol acetate for nutritional improvement in postoperative HNSCC undergoing radiotherapy focuses on appetite status, weight changes, lean body mass, inflammatory/nutritional markers, and quality of life, reflecting the broader impact of treatment (Liu et al., 2025).

#### 6.2.6 Regulatory pathways

The regulatory landscape for novel biomaterial-based drug delivery systems, especially those combining multiple drugs or gene therapies, is complex and evolving. These products often fall into hybrid categories, requiring extensive data on both the drug and the device components. Navigating the approval process, which demands rigorous preclinical testing, robust manufacturing controls, and well-designed clinical trials, can be a protracted and resource-intensive endeavor.

While preclinical research has unveiled a plethora of innovative biomaterial-based strategies for HNC, the journey to clinical translation is long and arduous. Addressing tumor heterogeneity, standardizing delivery methods, ensuring long-term biosafety, developing scalable manufacturing, and designing appropriate clinical endpoints are critical steps. The current evidence base, largely confined to HNSCC and OSCC in animal and in vitro models, highlights the need for continued investment in translational research to bridge the gap between laboratory promise and patient benefit.

## 7 Conclusion

HNC, particularly HNSCC and OSCC, presents a formidable challenge in oncology due to its complex local anatomy, the influence of mucosal and salivary barriers, the significant toxicity of conventional systemic therapies, the high risk of local recurrence, and the growing demand for sophisticated combination treatments. These inherent difficulties underscore the critical need for innovative drug delivery strategies that can overcome biological barriers, enhance drug accumulation at tumor sites, and minimize systemic side effects. Biomaterial-based delivery systems have emerged as a highly promising avenue to address these unmet clinical needs.

This comprehensive review has highlighted the remarkable progress in leveraging biomaterials for HNC treatment, organizing the discussion around key clinical application scenarios (Table S1). For localized/intratumoral delivery, nanoparticles (polymeric, lipid, inorganic, biomimetic, mesoporous silica, MOFs) and injectable hydrogels have demonstrated significant potential. These platforms enhance drug retention, enable controlled and stimuli-responsive release, and improve tissue penetration, leading to higher local drug concentrations and reduced systemic toxicity. Examples include redox-responsive micelles for selective doxorubicin release (Sun et al., 2018), iRGD-TRP-PK1-modified red blood cell vesicles for targeted chemotherapy (Bai et al., 2025), HA-altered gold complexes for pH and NIR dual-stimuli targeting (He et al., 2024a), and MMP-responsive hydrogels for sustained local chemotherapy (Li et al., 2019a; Wang et al., 2019a). These approaches offer a direct assault on the tumor while sparing healthy tissues.

For mucosal or patch-based delivery, particularly relevant for oral cancers, mucoadhesive hydrogels and patches are being developed to overcome salivary washout and mucosal barriers. By prolonging residence time and facilitating controlled release, these systems aim to improve local drug bioavailability. The multifunctional MOF-based nanohybrid, for instance, offers pH-responsive release for oral cancer therapy (Tan et al., 2020), while chitosan-based hydrogels show promise for localized chemo-photodynamic effects (Zhang et al., 2025c).

In the postoperative setting, localized controlled-release systems are crucial for preventing local recurrence. Injectable hydrogels and implantable scaffolds, capable of sustained drug release into the surgical bed, can eradicate residual cancer cells. The Dox/Cel/MOFs@Gel nanohybrid, for example, demonstrated potent antitumor activity and reduced systemic toxicity when used as an injectable implant (Tan et al., 2020), while bioactive sutures offer a novel approach for localized immune modulation (Shibuya et al., 2004).

Furthermore, biomaterials are proving instrumental in facilitating combination therapies with radiotherapy, chemotherapy, immunotherapy, and PDT. They enable the co-delivery of multiple agents, sensitize tumors to other treatments, and modulate the TME for synergistic effects. Notable advancements include chemo-gene co-delivery nanoparticles for metabolic reprogramming and immune activation (Zou et al., 2025), BSCgal for enhanced radio-immunotherapy (Jiang et al., 2025), zinc-mediated metalloimmunotherapy for OSCC (Zhou et al., 2025), and various light-responsive nanomedicines for combined photodynamic and photothermal therapies (Muhanna et al., 2015; Nam et al., 2018; Wang et al., 2019b). These multimodal strategies hold the key to overcoming drug resistance and improving therapeutic outcomes in HNC.

Despite these exciting developments, the translational challenges remain substantial. The current body of evidence is predominantly derived from in vitro and in vivo animal studies, primarily focusing on HNSCC and OSCC cell lines and xenograft models. Bridging the gap to clinical application is constrained by several critical factors: (1) Tumor heterogeneity: The vast genetic and phenotypic diversity within and between HNC tumors necessitates personalized approaches, making a “one-size-fits-all” biomaterial challenging. (2) Standardization of drug delivery: Achieving reproducible and consistent drug distribution, retention, and release in the complex human anatomy is difficult, especially for localized delivery. (3) Biosafety concerns: Long-term biocompatibility, biodegradability, and the potential for immunogenicity or toxicity of novel biomaterials and their degradation products require rigorous evaluation. (4) Scalable manufacturing processes: Translating laboratory-scale production of complex biomaterials into cost-effective, high-quality, and reproducible industrial manufacturing remains a significant hurdle. (5) Appropriate endpoint designs: Clinical trials need to move beyond traditional survival metrics to incorporate robust biomarkers and imaging techniques that can accurately assess local drug activity, tumor response, and reduction in treatment-related morbidity.

Research Gaps and Future Directions: Several critical research gaps need to be addressed to accelerate the clinical translation of biomaterial-based strategies for HNC. Firstly, there is a need for more sophisticated preclinical models that better recapitulate the human HNC microenvironment, including patient-derived organoids (Lin et al., 2024; Wong et al., 2024) and microphysiological systems (Yada et al., 2023), to provide more predictive efficacy and safety data. Secondly, a deeper understanding of the interactions between biomaterials, the TME, and the host immune system is essential to design more effective immunomodulatory and TME-reprogramming strategies. Thirdly, the development of smart, multi-stimuli-responsive biomaterials that can precisely adapt to dynamic changes within the tumor and its surroundings will enhance targeting and controlled release. Fourthly, integrating artificial intelligence and machine learning into biomaterial design, optimization, and manufacturing could significantly accelerate development and personalization (Miao et al., 2026). Finally, robust clinical trials, starting with early-phase studies focused on safety, pharmacokinetics, and pharmacodynamics, are urgently needed to validate the preclinical promise of these innovative biomaterial platforms in human HNC patients.

In conclusion, biomaterials represent a promising, yet still developing, approach for improving treatment delivery and multimodal therapy in head and neck cancer. While the preclinical evidence is compelling, overcoming the significant translational challenges will require concerted efforts from material scientists, oncologists, engineers, and regulatory bodies to bring these innovative solutions to the patients who desperately need them.

## CRediT authorship contribution statement

**Chang Li:** Writing – original draft, Visualization, Software, Resources, Project administration, Conceptualization. **Yuan Liu:** Writing – original draft, Visualization, Software, Resources, Formal analysis. **Zhifeng Wen:** Writing – original draft, Validation, Software, Formal analysis. **Junxiong Wen:** Writing – review & editing, Writing – original draft, Project administration, Investigation, Conceptualization. **Xiao Cui:** Writing – review & editing, Writing – original draft, Supervision, Conceptualization. **Xue Zhang:** Writing – review & editing, Writing – original draft, Supervision, Project administration, Investigation, Conceptualization.

## Ethics approval statement

Not applicable.

## Permission to reproduce material from other sources

Not applicable.

## Funding statement

There is no funding available to support this review.

## Declaration of competing interest

The authors declare that they have no known competing financial interests or personal relationships that could have appeared to influence the work reported in this paper

## Data Availability

No data was used for the research described in this article.
