# Supplementary material for: Biomaterial-based Drug delivery strategies for head and neck cancer: Local delivery, combination therapy, and translational challenges
Source: Int J Pharm X. 2026 Aug 26;12:100651. doi: 10.1016/j.ijpx.2026.100651 (PMC13545468; doi:10.1016/j.ijpx.2026.100651)
Supplement: Supplementary file 1 [file mmc1.docx]

**Table S1 Research Progresses on Local Administration and Combination Therapy Strategies of Biomaterials for HNC and OSCC.**

| **Nanoparticle or Biomaterial Components** | **Therapeutic Agents** | **Cancer Type / Experimental Model** | **Route / Mode of Local Administration** | **Targeting Strategy** | **Therapeutic Mechanism** | **Combination Treatment Modalities** | **Major Outcomes** | **Relevant References** |
| --- | --- | --- | --- | --- | --- | --- | --- | --- |
| ROS-responsive polymeric NPs (TPGS-PBTE) | Paclitaxel (PTX) | HNSCC / SCC-7 murine model | Intratumoral delivery | cRGD modification (active targeting) | ROS-responsive drug release; enhanced cytotoxicity and apoptosis | Chemotherapy | Enhanced tumor-targeted delivery; significant tumor growth inhibition in vivo | [23] |
| GSH-sensitive & FA-targeted NPs (FA-PEG-S-S-PCL) | Paclitaxel (PTX) | OSCC / HSC3 cells & murine model | Intratumoral delivery | Folic acid (FA) receptor targeting | GSH-sensitive release; FA-mediated endocytosis | Chemotherapy | Increased tumor accumulation; superior antitumor effects vs. free PTX | [25] |
| Lipid Nanoparticles (LNPHNSCC) | mRNA (PNP enzyme or p53 + CPX) | HNSCC & OSCC / In vivo solid tumors | Intratumoral / Local delivery | SORT (Selective Organ Targeting) | Tissue-specific mRNA delivery; TAM repolarization to M1 phenotype | Chemoimmunotherapy / Gene therapy | Preferential tumor delivery (liver sparing); anti-tumor response; effective in p53-resistant models | [27] |
| Aptamer-functionalized Liposomes | Imiquimod | HNC (HPV-related tongue cancer) | Local delivery | Aptamer active targeting | Selective cell uptake; immune activation via TLR pathways | Immunotherapy | Reduced viability/proliferation/migration; increased cell death | [32] |
| HA-altered Gold Complex (HA/Au-PDA@CZT) | Chemotherapeutic agents | HNSCC / In vivo model | Intratumoral delivery | Hyaluronic Acid (HA) + pH/NIR dual stimuli | Photothermal effect + pH-triggered drug release | Chemo-Photothermal Therapy (PTT) | High antitumor efficacy; low in vivo toxicity; significant tumor volume reduction | [35] |
| Multifunctional Gold Nanoplatform | DOX + PDPN Antibody | Oral Cancer / In vitro & In vivo | Intratumoral delivery | PDPN antibody active targeting | Chemo-PTT synergy; photothermal conversion | Chemo-Photothermal Therapy | Enhanced antitumor efficacy; high drug loading; low toxicity | [37] |
| MOF-based Nanohybrid (Dox/Cel/MOFs@Gel) | Doxorubicin (Dox) + Celecoxib (Cel) | OSCC / KB & SCC-9 cells & In vivo | Injectable implant / Mucosal | pH-responsive release | Synergistic chemo-anti-inflammatory effect; angiogenesis regulation | Combination Chemotherapy | Potent antitumor activity; reduced systemic toxicity; induced apoptosis | [11] |
| MMP-responsive Hydrogel (NDIMH) | Nano-Doxorubicin + ICG | HNSCC / SCC-15 & In vivo | Intratumoral injection | MMP-2 enzyme responsiveness | MMP-triggered sustained release; NIR-activated phototherapy | Chemo-Phototherapy | Synergistic antitumor efficacy; improved nanodrug retention; inhibited metastasis | [55] |
| Chitosan-ZnTAPP Hydrogel | Cisplatin (CDDP) | Solid Tumors (Lung/HNC relevant) | Local / Mucosal application | Photosensitizer integration | Singlet oxygen generation under irradiation | Photodynamic Therapy (PDT) + Chemotherapy | Significantly decreased IC50 for CDDP-resistant cells; overcomes resistance | [58] |
| Bioactive Sutures | IFN-γ, IL-2, Anti-CD3/CD28 | HNC / In vitro | Surgical site (Postoperative) | In situ immune modulation | Sustained Th1 immune response induction | Immunotherapy (Post-surgical) | Expanded flu-specific T cells; stimulated IL-12 secretion | [59] |
| Chemo-Gene Co-delivery NPs (PPPt IV) | Pt(IV) prodrug + CRISPR/Cas9-PKM2 | HNSCC / In vivo xenograft | Systemic / Tumor targeting | Metabolic reprogramming | DNA damage + PKM2 gene editing; reduced lactate/HIF-1α/PD-L1 | Chemo-Immunotherapy | Superior efficacy vs. cisplatin; activated T-cell immunity; reduced nephrotoxicity | [12] |
| Zn-Mediated Metalloimmunotherapy NPs (PYT@ZIF8) | Pyrithione + siRNA-SLC30A1 | OSCC / In vivo | Local / Tumor targeting | Zinc overload mechanism | Dual elimination of tumor & intratumoral bacteria; immune stimulation | Metalloimmunotherapy + PD-1 Blockade | Reprogrammed TME; hindered malignant transformation; synergistic with anti-PD-1 | [87] |
| Shikonin-loaded Lipid NPs (C-SNPs) | Shikonin (+ Mn2+) | HNSCC / In vivo | Systemic / Tumor targeting | CGKRK peptide modification | Necroptosis induction; cGAS-STING activation | Immunotherapy + PDT | Increased CD8+ T cell infiltration; sustained tumor suppression; sensitized "cold" tumors | [88] |
